# Lycium ruthenicum water extract preserves retinal ganglion cells in chronic ocular hypertension mouse models

**DOI:** 10.3389/fphar.2024.1404119

**Published:** 2024-07-03

**Authors:** Jinfeng Liu, Lina Zhou, Xueping Wu, Zihang Chen, Xiaofei Zheng, Huajun Wang, Kwok Fai So, Lan Ma, Jiantao Wang, Kin Chiu

**Affiliations:** ^1^ Shenzhen Eye Hospital, Shenzhen Eye Institute, Jinan University, Shenzhen, China; ^2^ Department of Ophthalmology, LKS Faculty of Medicine, The University of Hong Kong, Pokfulam, Hong Kong SAR, China; ^3^ Department of Laboratory Medicine, Peking University Shenzhen Hospital, Shenzhen, China; ^4^ Jinzhou Medical University, Jinzhou, China; ^5^ Department of Sports Medicine, The First Affiliated Hospital, Guangdong Provincial Key Laboratory of Speed Capability, The Guangzhou Key Laboratory of Precision Orthopedics and Regenerative Medicine, Jinan University, Guangzhou, China; ^6^ State Key Laboratory of Brain and Cognitive Sciences, The University of Hong Kong, Pokfulam, Hong Kong SAR, China; ^7^ Department of Psychology, The University of Hong Kong, Pokfulam, Hong Kong SAR, China; ^8^ Guangdong-Hongkong-Macau Institute of CNS Regeneration, Jinan University, Guangzhou, China; ^9^ Institute of Biopharmaceutical and Health Engineering, Tsinghua University Shenzhen Graduate School, Tsinghua University, Shenzhen, China; ^10^ Institute of Biomedical Health Technology and Engineering, Shenzhen Bay Laboratory, Shenzhen, China; ^11^ State Key Laboratory of Chemical Oncogenomics, Tsinghua Shenzhen International Graduate School, Tsinghua University, Shenzhen, China

**Keywords:** Lycium ruthenicum murray, glaucoma, retinal ganglion cell, oxidative stress, microglia

## Abstract

*Lycium ruthenicum* Murray (LR)*,* known as “black goji berry” or “black wolfberry”, is widely utilized in chinese herbal medicine. LR fruit showed its antioxidant and/or anti-inflammation activity in treating cardiac injury, experimental colitis, nonalcoholic fatty liver disease, fatigue, and aging. Glaucoma is the leading cause of irreversible blindness. Besides elevated intraocular pressure (IOP), oxidative stress and neuroinflammation were recognized to contribute to the pathogenesis of glaucoma. This study investigated the treatment effects of LR water extract (LRE) on retinal ganglion cells (RGCs) threatened by sustained IOP elevation in a laser-induced chronic ocular hypertension (COH) mouse model and the DBA/2J mouse strain. The antioxidation and anti-inflammation effects of LRE were further tested in the H_2_O_2_-challenged immortalized microglial (IMG) cell line *in vitro*. LRE oral feeding (2 g/kg) preserved the function of RGCs and promoted their survival in both models mimicking glaucoma. LRE decreased 8-hydroxyguanosine (oxidative stress marker) expression in the retina. LRE reduced the number of Iba-1+ microglia in the retina of COH mice, but not in the DBA/2J mice. At the mRNA level, LRE reversed the COH induced HO-1 and SOD-2 overexpressions in the retina of COH mice. Further *in vitro* study demonstrated that LRE pretreatment to IMG cells could significantly reduce H2O2 induced oxidative stress through upregulation of GPX-4, Prdx-5, HO-1, and SOD-2. Our work demonstrated that daily oral intake of LRE can be used as a preventative/treatment agent to protect RGCs under high IOP stress probably through reducing oxidative stress and inhibiting microglial activation in the retina.

## 1 Introduction

*Lycium ruthenicum* Murray (LR), also called “black goji berry”, or “black wolfberry”, has traditionally been utilized in medical practices to address conditions such as abnormal menopause, menstruation, and hypertension (Liu et al., 2020). LR fruit contains a rich assortment of compounds, such as anthocyanins, phenolic acids, polysaccharides, carotenoids, alkaloids, essential oils, and fatty acids. LR fruit has multifaceted functions including antioxidant, anti-fatigue, immune-enhancement, and anti-aging properties (Wang et al., 2018). LR extract (LRE) decreased the contents of lipid peroxidation and malondialdehyde (MDA) in serum and brain, accompanied by increased activities of superoxide dismutase (SOD) and glutathione peroxidase (GPX), in D-galactose induced aging mice (Cui et al., 2023). Polyphenols in LRE had neuroprotective effects against acrylamide-induced neurotoxicity (Pang et al., 2023). Polysaccharides in LRE protected cortical neurons against oxygen-glucose deprivation/reperfusion in neonatal hypoxic-ischemic encephalopathy (Deng et al., 2020).

Glaucoma is a leading cause of irreversible blindness, affecting an estimated 111.8 million people worldwide by 2040 (Allison et al., 2020). Progressive degeneration of retinal ganglion cells (RGCs) and subsequent visual field loss is the main symptom of glaucoma (Jayaram et al., 2023). Besides elevated intraocular pressure (IOP), mounting evidence suggests that additional mechanisms, such as oxidative stress and neuroinflammation, contribute to the pathogenesis and progression of glaucoma (Baudouin et al., 2021). In glaucoma, compromised retinal blood flow could trigger the generation of reactive oxygen species (ROS) in the retina (McMonnies, 2018; Wang et al., 2023). Elevated IOP can compress the optic nerve fiber and then reduce retrograde neurotrophin support for RGC axons, further contributing to ROS production in RGCs (McMonnies, 2018). Excessive ROS in RGCs directly induces their degeneration in glaucoma (Fernández-Albarral et al., 2024). ROS can also mediate microglial activation-related inflammation and neurotoxicity, which is a significant contributor to the pathogenesis of glaucoma (Baudouin et al., 2021). The interaction between oxidative stress and inflammatory response in microglia is known to contribute to its activation, playing a role in neurodegenerative diseases including glaucoma (Simpson and Oliver, 2020).

In this study, we investigated the effects of LRE on the retina in 2 mouse models mimicking glaucoma: a laser-induced chronic ocular hypertension (COH) mouse model and the DBA/2J mouse strain. Our primary objectives were to evaluate the impact of LRE on the function and survival of RGCs and to assess its ability to modulate oxidative stress and microglial activation under high IOP mimicking glaucoma.

## 2 Materials and methods

### 2.1 Preparation of *Lycium ruthenicum* water extract

*Lycium ruthenicum* water extract (LRE) was provided by Eu Yan Sang (HK) Ltd. LR from Qinghai, the People’s Republic of China, was used for this study. To prepare the LRE, 500 g dried LR was separated into 10 equal portions. Then, one portion of LR was put into 250 mL de-ionized water at 50°C–60°C for 15 min. Subsequently, LR was removed, and the extract was filtered. The filtrate was added with de-ionized water to make up to a final volume of 250 mL. This 250 mL filtrate was used to extract the next portion of LR as above procedures until all 10 portions were processed. Each milliliter of the final LRE contained 2 g of the crude drug. The LRE was stored in a refrigerator at 4°C.

### 2.2 Detection of anthocyanins and anthocyanidins in LRE

To test the anthocyanins and anthocyanidins contents, 39 g LRE was subjected to high-performance liquid chromatography (HPLC) (conducted by Eurofins Food Testing HK Ltd). The contents of anthocyanins and anthocyanidins are listed in Table 1. There were 0.0313% (w/w) anthocyanins and 0.0103% (w/w) anthocyanidins in the LRE. Delphinidine 3 glucoside was the major ingredient which was 0.027% (w/w) in the LRE and used as the standard for quality control.

**TABLE 1:** Identification of anthocyanins and anthocyanidins in LRE by HPLC.

Compound identity	Results	Unit
Delphinidin 3 galactoside	Not Detected	% (w/w)
Delphinidin 3 glucoside	0.027	% (ww)
Cyanidin 3 galactoside	Not Detected	% (ww)
Delphinidin 3 arabinoside	Not Detected	% (w/w)
Cyanidin 3 glucoside	Not Detected	% (w/w)
Petunidin 3 galactoside	Not Detected	% (ww)
Cyanidin 3 arabinoside	0.00165	% (w/w)
Petunidin 3 glucoside	Not Detected	% (w/w)
Delphinidin	Not Detected	% (w/w)
Peonidin 3 galactoside	Not Detected	% (w/w)
Petunidin 3 arabinoside	0.0019	% (ww)
Peonidin 3 glucoside	Not Detected	% (ww)
Malvidin 3 galactoside	Not Detected	% (w/w)
Peonidin 3 arabinoside	Unable to determine due to interference	% (w/w)
Malvidin 3 glucoside	Not Detected	% (ww)
Cyanidin	0.00934	% (w/w)
Malvidin 3 arabinoside	0.000773	% (w/w)
Petunidin	0.000647	% (w/w)
Peonidin	0.000141	% (w/w)
Malvidin	0.000131	% (ww)
Total anthocyanins	0.0313	% (w/w)
Total anthocyanidins	0.0103	% (w/w)
Total anthocyanins and anthocyanidins	0.0416	% (w/w)

### 2.3 Animals

CX3CR-1^GFP^ knock-in/knock-out mice (Jackson Laboratory, stock No. 005582), DBA/2J mice (Jackson Laboratory, stock No. 000671), and C57BL/6J mice were obtained from the Laboratory Animal Unit of the University of Hong Kong. The mice were housed in a controlled environment with a 12-h light/dark cycle, maintaining a pathogen-free setting. All animal procedures were conducted in accordance with the ARRIVE guidelines and approved by the Committee on the Use of Live Animals in Teaching and Research of the University of Hong Kong.

### 2.4 Laser photocoagulation induced COH mouse model

COH mouse model was constructed by laser photocoagulation on the corneal limbus according to an optimized protocol (Feng et al., 2013). Briefly, female CX3CR1^+/GFP^ mice at the age of 6 months were anesthetized by intraperitoneal injection with a mixture of ketamine (80 mg/kg) and xylazine (8 mg/kg). The right eyes were applied with 1% cyclopentolate hydrochloride (Mydriacyl, Alcon Labs, Inc., Fort Worth, TX, USA) for pupil dilation followed by proparacaine hydrochloride (0.5% alcaine, Alcon) for topical anesthesia. The anterior chamber was punctured with a 30 G syringe needle to drain the aqueous humor. Subsequently, 60–80 consecutive laser spots (Size, 500 μm; power, 800 mW; pulse duration, 50 msec) were delivered perpendicularly to the limbus surface, while sparing the nasal area, using a 532 nm laser (Lumenis Novus Spectra, Yokneam, Israel). About 10% of mice eyes exhibiting anterior chamber hemorrhage, cataract, or corneal ulcer following the induction of IOP elevation were excluded from the study.

### 2.5 Measurement of IOP

The IOP of mouse eyes was measured using a rebound tonometer (Icare®TonoLab, Colonial Medical Supply, Franconia, NH). In the laser-induced COH model, IOP measurements were performed on awake mice without any eye drops, whereas measurements were conducted on general anesthetized DBA/2J mice under local corneal anesthesia. Each IOP value was determined by averaging six consecutive measurements and the IOP level of the mouse eye was represented by the average of three such values.

### 2.6 Animal feeding and grouping

The dose of LRE oral feeding at 2 g/kg body weight was chosen according to the previous LRE dose-response study in mice suffered radiation injury (Duan et al., 2015). For the laser-induced COH mice, daily LRE oral feeding started at 7 days before laser photocoagulation till 30 days after the induction of COH. Distilled water was fed as a placebo control. At the end of the experiment, there were 22 normal control eyes, 20 eyes with COH fed with water, and 22 eyes with COH fed with LRE for data analysis. For DBA/2J mice, daily LRE feeding started at 6 months of age for 4 months and ended at 10 months of age. C57BL/6J mice at 10 months of age were used as wild-type controls. There were 10 mice in each group, both eyes of the DBA/2J and C57BL/6J mice were used for analysis.

### 2.7 Flash electroretinography (ERG)

The function of RGCs was evaluated using flash ERG, following the standard protocol of the International Society for Clinical Electrophysiology of Vision. Following general anesthesia, 1% cyclopentolate hydrochloride (Mydriacyl, Alcon) was applied to the eyes to dilate the pupil and then proparacaine hydrochloride (0.5% alcaine, Alcon) was applied as topical anesthesia for 5 min. Afterward, ERG signals were recorded using an ERG system (Espion E2 Electrophysiology System, Diagnosys LLC, USA). Full-field flash ERG test was conducted at a photopic intensity of 3.0 and 10.0 cd s.m^-2^ to detect the photopic negative response (PhNR) which represents the RGCs activity. The acquired data were analyzed using Axon pCLAMP 10 software (Molecular Devices Corp., Sunnyvale, CA, USA).

### 2.8 Retinal ganglion cell counting on flat-mounted retina

In the COH mice study, the RGC survival was evaluated by counting the number of Brn-3a+ cells on the flat-mounted retina. Mice were euthanized, and the eyeballs were enucleated and fixed in 4% paraformaldehyde (PFA) for 1 h. Subsequently, the retina was dissected from the sclera and flat mounted with the RGC layer faceup under a stereo microscope. To stain the RGCs, the retinas were rinsed with PBS, followed by blocking with a solution containing 10% normal donkey serum and 0.1% Triton X-100 in PBS. The retinas were then incubated overnight at 4°C with goat anti-Brn-3a (1:500, Santa Cruz, Dallas, USA). After thorough washing, the retinas were incubated with a secondary antibody, Alexa-568 fluorescent-conjugated donkey anti goat IgG secondary antibody (1:500; Thermo Fisher Scientific, Waltham, MA, USA), for 2 h at room temperature. To visualize cell nuclei, the retinas were counterstained with 4’,6-Diamidino-2- phenylindole (DAPI) (1:1000). Confocal images were acquired using a ZEISS LSM 800 confocal microscope (Carl Zeiss Microscopy GmbH, Germany), and the number of Brn-3a+ cells was quantified using ImageJ software (National Institutes of Health, Bethesda, Maryland, USA).

### 2.9 Retinal section histological analysis

In the DBA/2J mice study, the RGC survival was evaluated by counting the nuclei in the RGC layer. After standard eyeball fixation, dehydration, and paraffin embedding, retinal cross sections containing optic nerve were collected for further analysis. These sections were stained with hematoxylin and eosin (H&E) and mounted using DPX mounting medium. Images were captured using an optical microscope (Nikon Eclipse 80i, Tokyo, Japan). The number of nuclei in the RGC layer was quantified using ImageJ software.

### 2.10 Immunohistochemical staining

The eye sections were deparaffinized and subjected to antigen retrieval by immersing them into 95 °C citric acid buffer for 15 min. Subsequently, the sections were blocked with 10% normal goat serum in PBS and then incubated overnight at 4°C with primary antibodies, including rabbit anti-Iba-1 (1:500, WAKO, Chou-ku, Osaka, Japan), rabbit anti-8-hydroxyguanosine (8-OHdG) (1:500, Abcam, Cambridge, UK), and rabbit anti-caspase3 (1:500, Abcam). Following that, the sections were incubated with Alexa-568 or 488 fluorescent-conjugated goat anti rabbit IgG secondary antibodies (1:500; Thermo Fisher Scientific) for 1 h and then counterstained with DAPI (1:1000) at room temperature. Images were captured using a ZEISS LSM 800 confocal microscope. The number of positive cells and fluorescence intensity were quantified using ImageJ software.

### 2.11 Cell culture and treatment

The immortalized microglial (IMG) cell line (Cat. SCC134, Sigma-Aldrich, Burlington, MA, USA) was cultured in Dulbecco’s Modified Eagle Medium (DMEM) with high glucose (Thermo Fisher Scientific) supplemented with 10% fetal bovine serum (FBS) (Invitrogen, Carlsbad, CA, USA). The cells were maintained at 37°C in a humidified atmosphere containing 95% air and 5% CO2. To prevent contamination, 1% Penicillin-Streptomycin (Thermo Fisher Scientific) was added to the medium.

For the experiments, IMG cells were seeded in 96-well plates (1 × 10^4^ cells/well) or 12-well plates (1.5 × 10^5^ cells/well) in DMEM with high glucose supplemented with 1% FBS and allowed to incubate overnight before the treatments. To test the dose-response cytotoxicity (LDH test) induced by H2O2 or LRE, cells were treated with 50, 100, 200, 300, 400, and 800 µM of H2O2 (Merck Millipore, Burlington, MA, USA) or 100, 200, 400, and 800 μg/mL of LRE for 24 h followed by LDH test. The antioxidant effect of LRE was first evaluated in the pretreatment test. The IMG cells were incubated with LRE for 2 h at 10, 50, 100, and 200 μg/mL before 300 µM H2O2 stress. In the further experiments, the timing for 200 μg/mL LRE to be applied were evaluated 2 h before (pretreatment), at the same time (simultaneous), or 2 h after (post-treatment) the H2O2 stimulation in the IMG cells. To explore the mechanism of LRE antioxidative effect, the expressions of H2O2 decomposition related enzymes including catalase, GPX-1, GPX-4, peroxiredoxin (Prdx)-1, Prdx-2, Prdx-3, Prdx-4, Prdx-5, and Prdx-6 were detected at 2 h after 200 μg/mL LRE treatment in IMG cells. Furthermore, LRE pretreatment effect on the antioxidant genes such as heme oxygenase (HO)-1 and SOD-2 were evaluated. Untreated cells were applied as controls. Each experiment was repeated three times.

### 2.12 Cytotoxicity assay

The cytotoxicity of IMG cells was assessed using a Pierce LDH Cytotoxicity Assay Kit (Cat. 88953, Thermo Fisher Scientific). Cells were seeded in a 96-well plate. After 24 h of treatment with H2O2 and/or LRE, the cell culture supernatant was collected and mixed with LDH assay buffer in a new 96-well plate. After incubation for 30 min, the reaction was stopped by adding the stop solution to the sample wells. The absorbance at 490 nm and 680 nm was measured using a spectrometry (EnSpire Multimode Plate Reader, PerkinElmer, Waltham, MA, USA). The LDH activity was determined by subtracting the absorbance at 680 nm from the absorbance at 490 nm.

### 2.13 Quantitative reverse transcription polymerase chain reaction

Total RNA from mouse retinas or IMG cell samples was extracted using an RNA extraction kit (Cat. 74106, QIAGEN, Hilden, Germany). Subsequently, the RNA was reverse transcribed into cDNA using a reverse transcription kit (Cat. 205413, QIAGEN). The resulting cDNA samples were then subjected to real-time PCR analysis using an SYBR Green PCR Kit (Cat. 208056, QIAGEN). The amplification process consisted of an initial incubation at 95 °C for 2 min, followed by 40 cycles of denaturation at 95 °C for 5 s and annealing at 60 °C for 15 s. Melting curve analysis was performed to ensure amplification specificity. The primer sequences of the tested genes are listed in Table 2. The gene expression levels of target genes were normalized to housekeeping gene β-actin. The 2^−ΔΔCT^ formula was applied for calculation purposes.

**TABLE 2:** Primer sequences used for real-time PCR.

Gene	Forward primer (5′-3′)	Reverse primer (5′-3′)	Reference
β-actin	GTG​ACG​TTG​ACA​TCC​GTA​AAG​A	GCC​GGA​CTC​ATC​GTA​CTC​C	NM_007393
IL-1β	CTG​TGA​CTC​ATG​GGA​TGA​TGA​TG	CGG​AGC​CTG​TAG​TGC​AGT​TG	NM_008361
IL-6	CTG​CAA​GAG​ACT​TCC​ATC​CAG	AGT​GGT​ATA​GAC​AGG​TCT​GTT​GG	NM_031168
IL-10	CTT​ACT​GAC​TGG​CAT​GAG​GAT​CA	GCA​GCT​CTA​GGA​GCA​TGT​GG	NM_010548
CX3CR1	GAG​TAT​GAC​GAT​TCT​GCT​GAG​G	CAG​ACC​GAA​CGT​GAA​GAC​GAG	NM_009987
HO-1	GAT​AGA​GCG​CAA​CAA​GCA​GAA	CAG​TGA​GGC​CCA​TAC​CAG​AAG	NM_010442
SOD-2	CAG​ACC​TGC​CTT​ACG​ACT​ATG​G	CTC​GGT​GGC​GTT​GAG​ATT​GTT	NM_013671
Catalase	AGC​GAC​CAG​ATG​AAG​CAG​TG	TCC​GCT​CTC​TGT​CAA​AGT​GTG	NM_009804
GPX-1	AAT​GTC​GCG​TCT​CTC​TGA​GG	TCC​GAA​CTG​ATT​GCA​CGG​G	NM_008160
GPX-4	GAT​GGA​GCC​CAT​TCC​TGA​ACC	CCC​TGT​ACT​TAT​CCA​GGC​AGA	NM_008162
Prdx-1	AAT​GCA​AAA​ATT​GGG​TAT​CCT​GC	CGT​GGG​ACA​CAC​AAA​AGT​AAA​GT	NM_011034
Prdx-2	CAC​CTG​GCG​TGG​ATC​AAT​ACC	GAC​CCC​TGT​AAG​CAA​TGC​CC	NM_011563
Prdx-3	GGT​TGC​TCG​TCA​TGC​AAG​TG	CCA​CAG​TAT​GTC​TGT​CAA​ACA​GG	NM_007452
Prdx-4	CTC​AAA​CTG​ACT​GAC​TAT​CGT​GG	CGA​TCC​CCA​AAA​GCG​ATG​ATT​TC	NM_016764
Prdx-5	GGC​TGT​TCT​AAG​ACC​CAC​CTG	GGA​GCC​GAA​CCT​TGC​CTT​C	NM_012021
Prdx-6	CGC​CAG​AGT​TTG​CCA​AGA​G	TCC​GTG​GGT​GTT​TCA​CCA​TTG	NM_007453

GPX, glutathione peroxidase; HO-1, heme oxygenase 1; Prdx, peroxiredoxin; SOD-2, superoxide dismutase 2

### 2.14 Statistical analysis

GraphPad Prism 8.0 software (GraphPad software, San Diego, California, USA) was utilized to create statistical graphs. Data analysis was performed using SPSS software for Windows (version 20.0; SPSS, Inc., IL, USA). To compare multiple groups, a one-way ANOVA was conducted, followed by Fisher’s Least Significant Difference (LSD) test for multiple comparisons or Dunnett’s test when the variances of all groups were not equal. When comparing two groups, two-tailed Student’s t-tests were employed. The data was presented as mean ± SD, and a significance level of *p < 0.05 was considered statistically significant.

## 3 Results

### 3.1 LRE preserved retinal function under sustained IOP elevation

**FIGURE 1:**
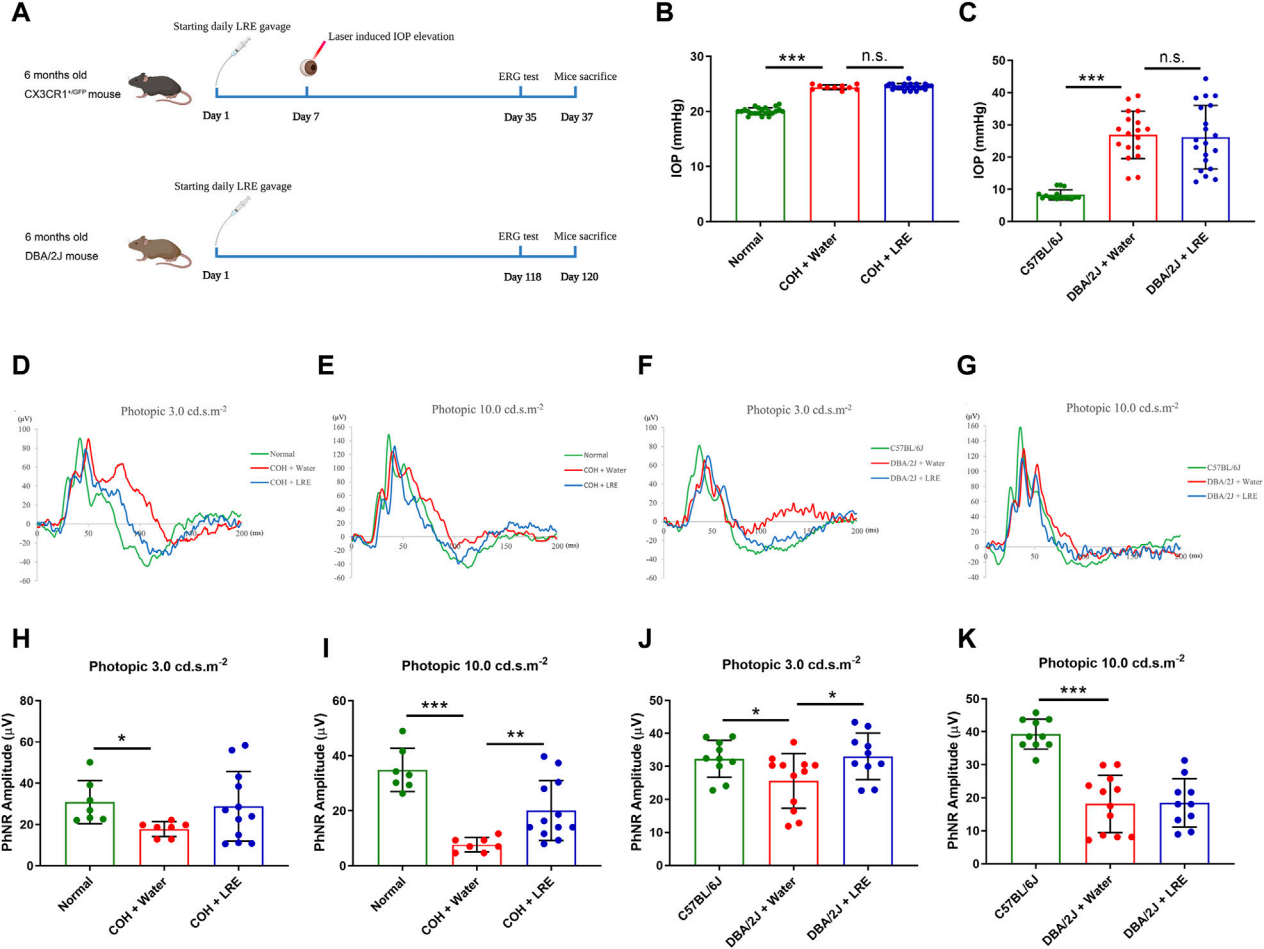
LRE preserved RGC function in the eyes of the laser-induced COH and DBA/2J mice without affecting the elevated IOP. **(A)** A schematic representation of the experimental flows in the laser-induced COH mouse model (upper lane) and the DBA/2J mouse strain (lower lane) (Diagram is created in BioRender.com). **(B)** IOP measurement on the awake mice in the laser-induced COH mice after 30 days of laser photocoagulation. **(C)** IOP measurement on the anesthetized C57BL/6J and DBA/2J mice at the age of 10 months. **(D–G)** Representative ERG waves were shown on the assessment of RGC function using a photopic ERG test with flash strengths of 3.0 and 10.0 cd s.m^-2^ in the laser-induced COH, DBA/2J, and control eyes after LRE treatment. **(H–K)** Bar chart figures demonstrated that LRE oral feeding significantly increased the PhNR amplitudes in the COH eyes at photopic 10.0 cd s.m^-2^ and in the DBA/2J mice eyes at photopic 3.0 cd s.m^-2^. *, *p* < 0.05; **, *p* < 0.01; ***, *p* < 0.001; n. s., not significant.

Daily feeding of LRE (2 g/kg) was kept till 30 days after COH induction and started from 6 months till 10 months of age in the DBA/2J mice (Figure 1A). At 30 days after COH induction, the awake IOP in the normal control mice was 20.05 ± 0.64 mmHg. There was significant IOP elevation in both water-fed (24.40 ± 0.44 mmHg, ***p < 0.001) and LRE-fed (24.52 ± 0.56 mmHg, ***p < 0.001) COH eyes. There was no significant IOP change between LRE- and water-fed groups (Figure 1B). In the DBA/2J study, the IOP in C57BL/6J mice was 8.26 ± 1.53 mmHg under general anesthesia at 10 months of age. There was significant IOP elevation in the DBA/2J mice reaching about 26 mmHg (***p < 0.001, Figure 1C). There was no significant change between the water- (26.89 ± 7.36 mmHg) and LRE-fed (26.17 ± 9.85 mmHg) eyes.

Retinal function was evaluated by the photopic ERG test, there was a significant reduction in the PhNR amplitude in the COH eyes. At photopic 3.0 cd s.m^-2^, it reduced from 30.86 ± 10.50 (normal) to 17.84 ± 3.61 μV (*p = 0.044, Figures 1D,H). At photopic 10 cd s.m^-2^, it reduced from 34.85 ± 7.88 (normal) to 7.67 ± 2.60 μV (***p < 0.001, Figure 1E, I). LRE oral feeding significantly increased the PhNR amplitude in the COH eyes to 20.12 ± 10.92 μV (**p = 0.007 vs. water feeding) at photopic 10 cd s.m^-2^ (Figure 1E, I). In the DBA/2J mice at age of 10 months, the PhNR amplitude significantly reduced from 32.33 ± 5.62 (C57BL/6J) to 25.62 ± 8.31 μV at photopic 3.0 (*p = 0.037, Figure 1F, J), and from 39.29 ± 4.56 (C57BL/6J) to 18.16 ± 8.67 μV (***p < 0.001, Figure 1G, K) at photopic 10.0 cd s.m^-2^. Unlike in the COH model, the significantly increased PhNR amplitude was detected at the photopic intensity of 3.0 cd s.m^-2^, reaching 33.03 ± 7.06 μV (*p = 0.022 vs. water feeding) after 4 months of LRE oral feeding (Figure 1F, J).

### 3.2 LRE prevented RGC loss induced by sustained IOP elevation

**FIGURE 2:**
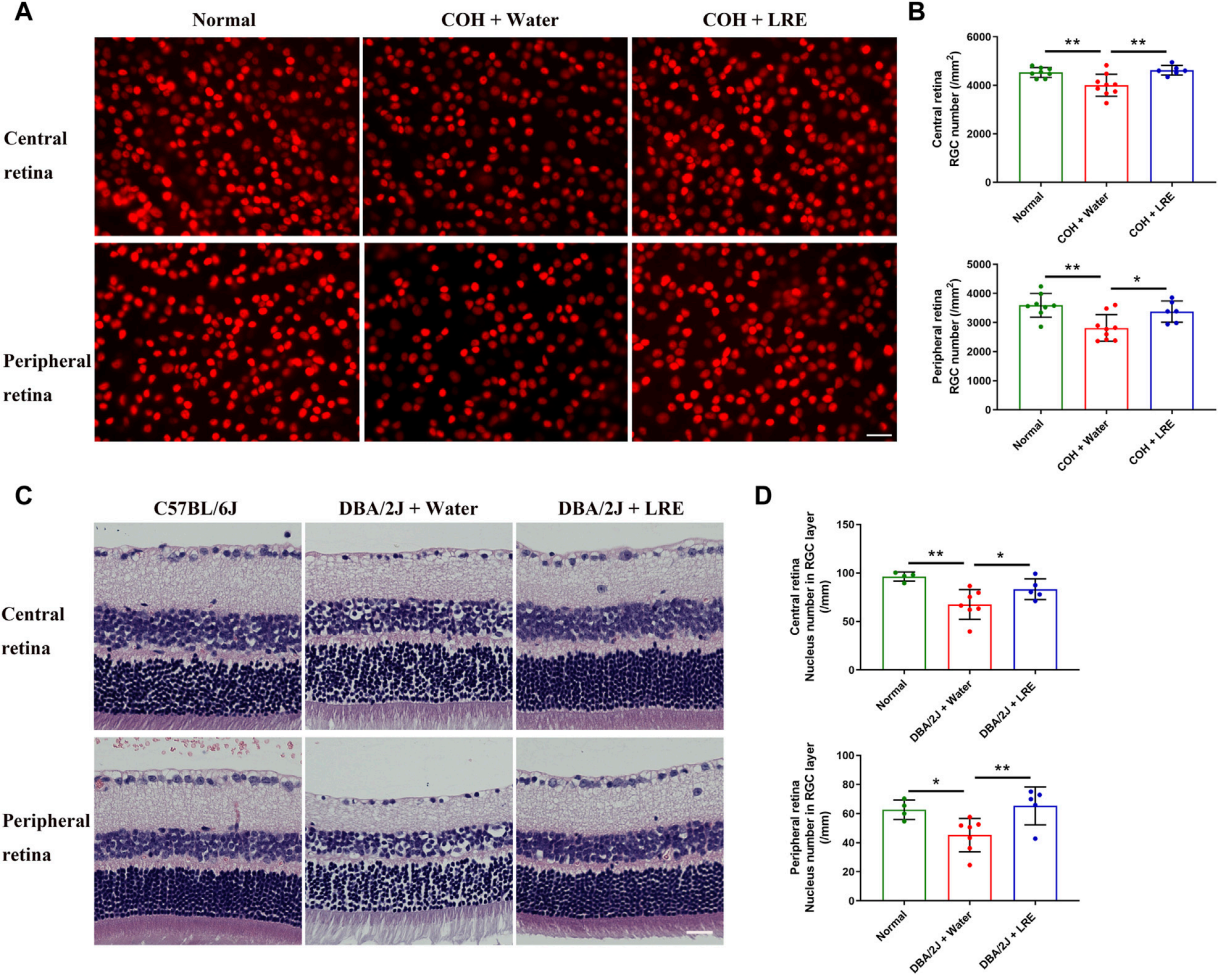
LRE promoted RGC survival in the eyes of the laser-induced COH and DBA/2J mice. **(A)** Representative central retina (upper lane) and peripheral retina (lower lane) images of Brn-3a stained RGCs (red) in the retinal flat-mounts from the normal, water-fed, and LRE-fed COH mice. The density of RGCs markedly reduced in the water-fed COH eyes (middle column). Scale bar, 25 µm. **(B)** 30 days of LRE oral feeding significantly increased the RGC number in the central and peripheral retina of the COH eyes. **(C)** Representative images of H&E-stained retinal sections from the C57BL/6J control, water-fed, and LRE-fed DBA/2J mice. There were fewer cells in the RGC layer in the water-fed DBA/2J mice both at the central and peripheral retina. Scale bar, 25 µm. **(D)** 4 months of LRE oral feeding significantly increased nucleus density in the RGC layer of both the central and peripheral retina of the DBA/2J mice. *, *p* < 0.05; **, *p* < 0.01.

**FIGURE 3:**
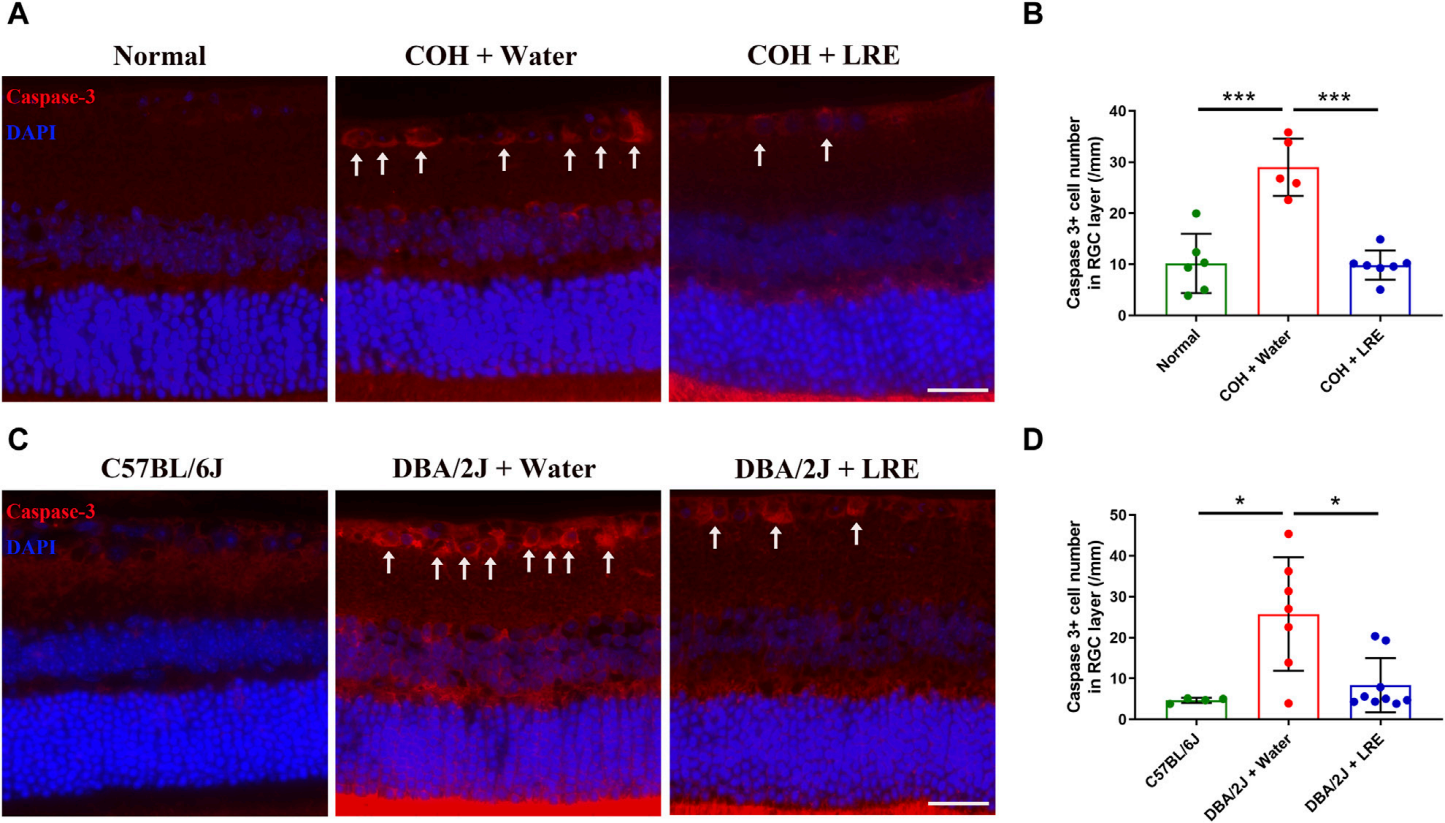
LRE inhibited apoptosis in the RGC layer of the laser-induced COH and DBA/2J mice. **(A)** Representative images of caspase-3 positive cells (red, arrows) in the retinal sections from the normal, water-fed, and LRE-fed COH mice. Scale bar, 25 µm. **(B)** LRE significantly decreased the number of caspase-3 positive cells in the RGC layer of the COH eyes. **(C)** Representative images of caspase-3 positive cells (red, arrows) in the retinal sections from the C57BL/6J control, water-fed, and LRE-fed DBA/2J mice. Scale bar, 25 µm. **(D)** LRE significantly decreased the number of caspase-3 positive cells in the RGC layer of the DBA/2J mice. *, *p* < 0.05; ***, *p* < 0.001.

In the COH study, the RGC survival was evaluated by counting the Brn-3a+ cells in the flat-mounted retina (Figure 2A). 30 days of elevated IOP induced significant loss of RGC survival (**p = 0.004), it reduced from 4,530 ± 203 in the normal control eyes to 4,008 ± 456 cells/mm^2^ in the water-fed COH mice at the central retina. There was even severer RGC loss at the peripheral retina, it reduced from 3,592 ± 412 to 2,815 ± 457 cells/mm^2^ (**p = 0.001, normal vs. water-fed COH mice) (Figure 2B). The LRE oral feeding started 7 days before laser induced IOP elevation till 30 days after COH established. LRE significantly reduced the RGC loss, the RGC number reached 4,621 ± 200 (**p = 0.002) in the central retina and 3,377 ± 368 cells/mm^2^ (*p = 0.02) in the peripheral retina (Figure 2B). The apoptotic cell marker, Caspase-3, was detected in the retinal cross sections (Figure 3A). In the RGC layer, Caspase-3 positive cell number significantly increased in water-fed COH mice (29 ± 6 cells/mm, ***p < 0.001) comparing to the controls (10 ± 6 cells/mm) and LRE significantly reduced it to 10 ± 3 cells/mm (***p < 0.001 vs. water feeding, Figure 3B).

This neuroprotective effect of LRE was further proved in the congenital glaucoma model, DBA/2J mice. In the retinal cross sections (Figure 2C), the number of cells in the RGC layer markedly reduced from 96 ± 5 (C57BL/6J mice) to 68 ± 15/mm (**p = 0.001, Figure 2D) in the DBA/2J mice with water feeding at the central retina. 4 months of LRE treatment significantly preserved cells to a density of 83 ± 11/mm (*p = 0.046, LRE vs. water feeding). A similar trend was observed in the peripheral retina, cells reduced from 63 ± 7 (C57BL/6J mice) to 45 ± 11/mm (*p = 0.027, Figure 2D) in the water-fed and then increased to 65 ± 13/mm (**p = 0.009, LRE vs. water feeding) in the LRE-fed DBA/2J mice. The apoptotic caspase-3+ cell number was significantly increased in the RGC layer in DBA/2J mice (26 ± 14 cells/mm) compared to the C57BL/6J controls (5 ± 1 cells/mm, *p = 0.019, Figure 3C, D). LRE significantly reduced the apoptotic cell number to 8 ± 7 cells/mm (*p = 0.043, LRE vs. water feeding, Figure 3D).

### 3.3 LRE reduced oxidative stress in the mouse retina exposed to sustained IOP elevation

**FIGURE 4:**
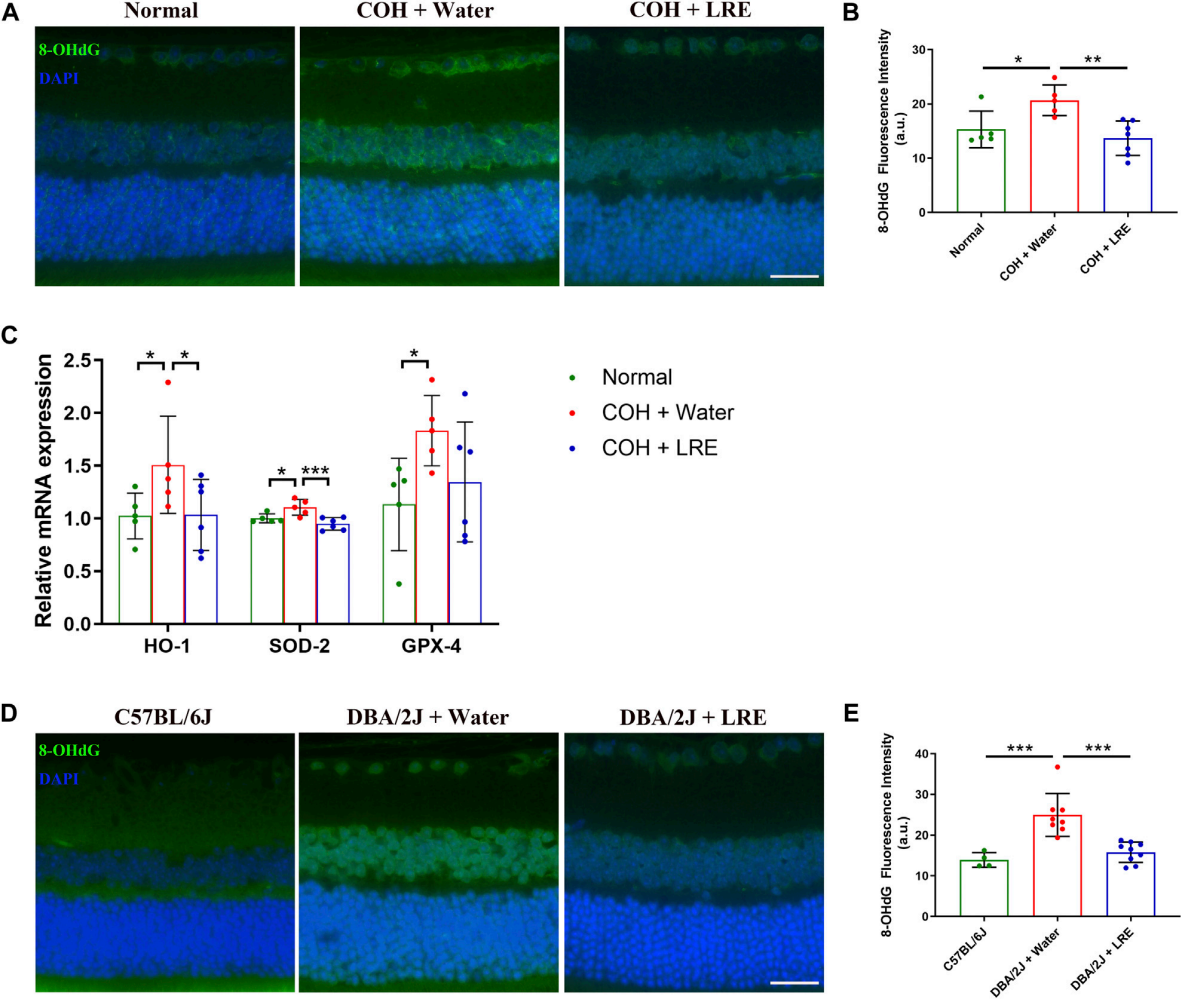
LRE mitigated oxidative stress in the retina of the laser-induced COH and the DBA/2J mice. **(A)** Representative images of 8-OHdG stained retinal sections from normal, water-fed, and LRE-fed COH mice. Scale bar, 25 µm. **(B)** LRE significantly reduced the fluorescence intensity of 8-OHdG in the retina of the COH eyes. **(C)** The gene expressions of HO-1, SOD-2, and GPX-4 in the retina of the normal, water-fed, and LRE-fed COH eyes. **(D)** Representative images of 8-OHdG stained retinal sections from the C57BL/6J control, water-fed, and LRE-fed DBA/2J mice. Scale bar, 25 µm. **(E)** LRE significantly reduced the fluorescence intensity of 8-OHdG in the retina of the DBA/2J mice. *, *p* < 0.05; **, *p* < 0.01; ***, *p* < 0.001.

Guanine in DNA is converted to 8-OHdG upon free radical attack under oxidative stress (Andrés et al., 2023). Compared to control retinas, the fluorescent intensity of the 8-OHdG markedly increased in water-fed COH eyes in the inner retina including RGC layer and the inner nuclear layer (INL), and slightly upregulated in the outer nuclear layer (ONL) (Figure 4A). Semi-quantitative analysis demonstrated a significant increase in 8-OHdG level in the retina of water-fed COH mice (*p = 0.018 vs. normal), which was effectively reduced by LRE treatment (**p = 0.002, Figure 4B). Furthermore, the gene expressions of HO-1, SOD-2, and GPX-4 significantly increased by 1.51 ± 0.46 (*p = 0.049), 1.10 ± 0.07 (*p = 0.017), and 1.83 ± 0.33 folds (*p = 0.033), respectively, in the retina of COH mice (Figure 4C). LRE mitigated the tissue response to oxidative stress in the retina of the COH eyes. LRE oral feeding reduced the changes of HO-1, SOD-2, and GPX-4 to 1.03 ± 0.34 (*p = 0.044 vs. water feeding), 0.95 ± 0.06 (***p < 0.001 vs. water feeding), and 1.35 ± 0.57 folds (*p* = 0.108 vs. water feeding), respectively in the COH eyes.

The antioxidant properties of LRE treatment were further investigated in DBA/2J mice. Similar to the changes in COH eyes, there was increased 8-OHdG expression in the inner retina of water-fed DBA/2J eyes (Figure 4D). Semi-quantification revealed a significant increase in 8-OHdG expression in the retina of water-fed DBA/2J mice compared to the C57BL/6J mice (***p < 0.001, Figure 4E), whereas LRE oral feeding significantly reduced 8-OHdG level in the retina of DBA/2J mice (***p < 0.001, Figure 4E).

### 3.4 LRE inhibited microglial activation in the mouse retina under sustained IOP elevation

**FIGURE 5:**
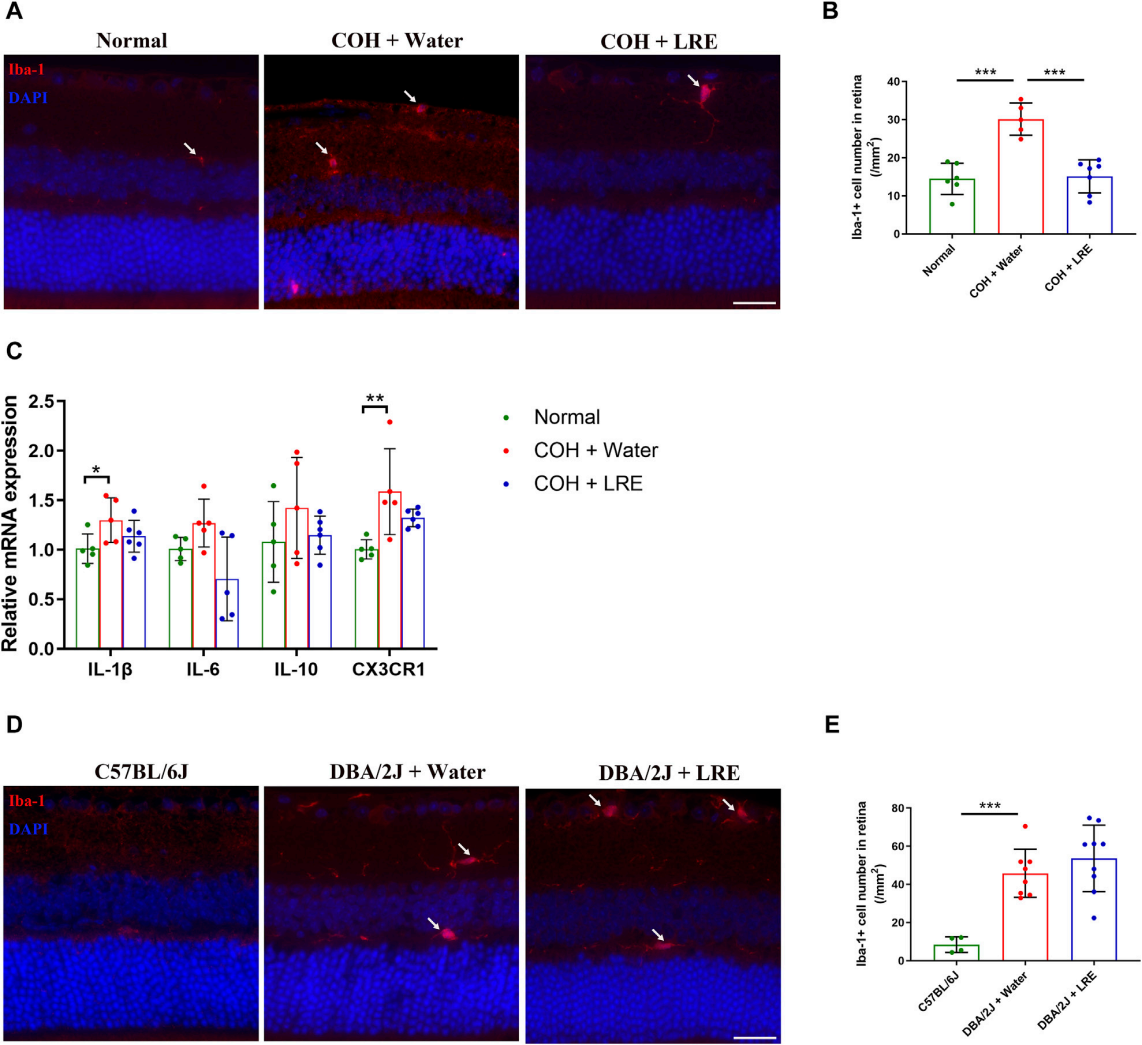
LRE affected microglial activation in the retina of the laser-induced COH eyes. **(A)** Representative images of Iba-1 positive cells (red, arrows) in the retinal sections from the normal, water-fed, and LRE-fed COH mice. Scale bar, 25 µm. **(B)** LRE significantly decreased the number of Iba-1 positive microglia in the retina of COH eyes. **(C)** The gene expressions of IL-1β, IL-6, IL-10, and CX3CR1 in the retina of normal, water-fed, and LRE-fed COH eyes. **(D)** Representative images of Iba-1 positive cells (red, arrows) in the retinal sections from the C57BL/6J, water-fed, and LRE-fed DBA/2J mice. Scale bar, 25 µm. **(E)** LRE did not change the Iba-1 positive microglial cell number in the retina of DBA/2J mice. *, *p* < 0.05; **, *p* < 0.01; ***, *p* < 0.001.

Microglial activation in the retina of laser-induced COH mice was evaluated by quantifying the number of Iba-1+ microglia in the retinal sections. The microglial number was significantly increased from 14 ± 4 (normal) to 30 ± 4 cells/mm^2^ (***p < 0.001) in the retina of water-fed COH mice (Figure 5A, B). LRE treatment significantly reduced the microglial number to 15 ± 4 cells/mm^2^ (***p < 0.001 vs. water feeding) (Figures 5A, B). The gene expressions of IL-1β, IL-6, IL-10, and CX3CR1 in the water-fed COH mice retina was increased by 1.30 ± 0.23, 1.27 ± 0.24, 1.42 ± 0.51, and 1.59 ± 0.43 folds (vs. normal), respectively. There was significant elevation in IL-1β (*p = 0.025) and CX3CR1 (**p = 0.003, Figure 5C) in the water-fed COH eyes comparing to normal eyes. LRE oral feeding reduced COH elevated IL-1β, IL-6, IL-10, and CX3CR1 levels without reaching statistical significance (Figure 5C).

In the retina of 10 months old water-fed DBA/2J mice, the Iba-1+ microglial number was significantly increased to 46 ± 13 cells/mm^2^ (vs. C57BL/6J, ***p < 0.001). 4 months of LRE oral feeding did not significantly affect the microglial number (54 ± 17 cells/mm^2^) in the retina (Figure 5D, E).

### 3.5 LRE upregulated antioxidant enzymes in microglial cells under H2O2 stress

**FIGURE 6:**
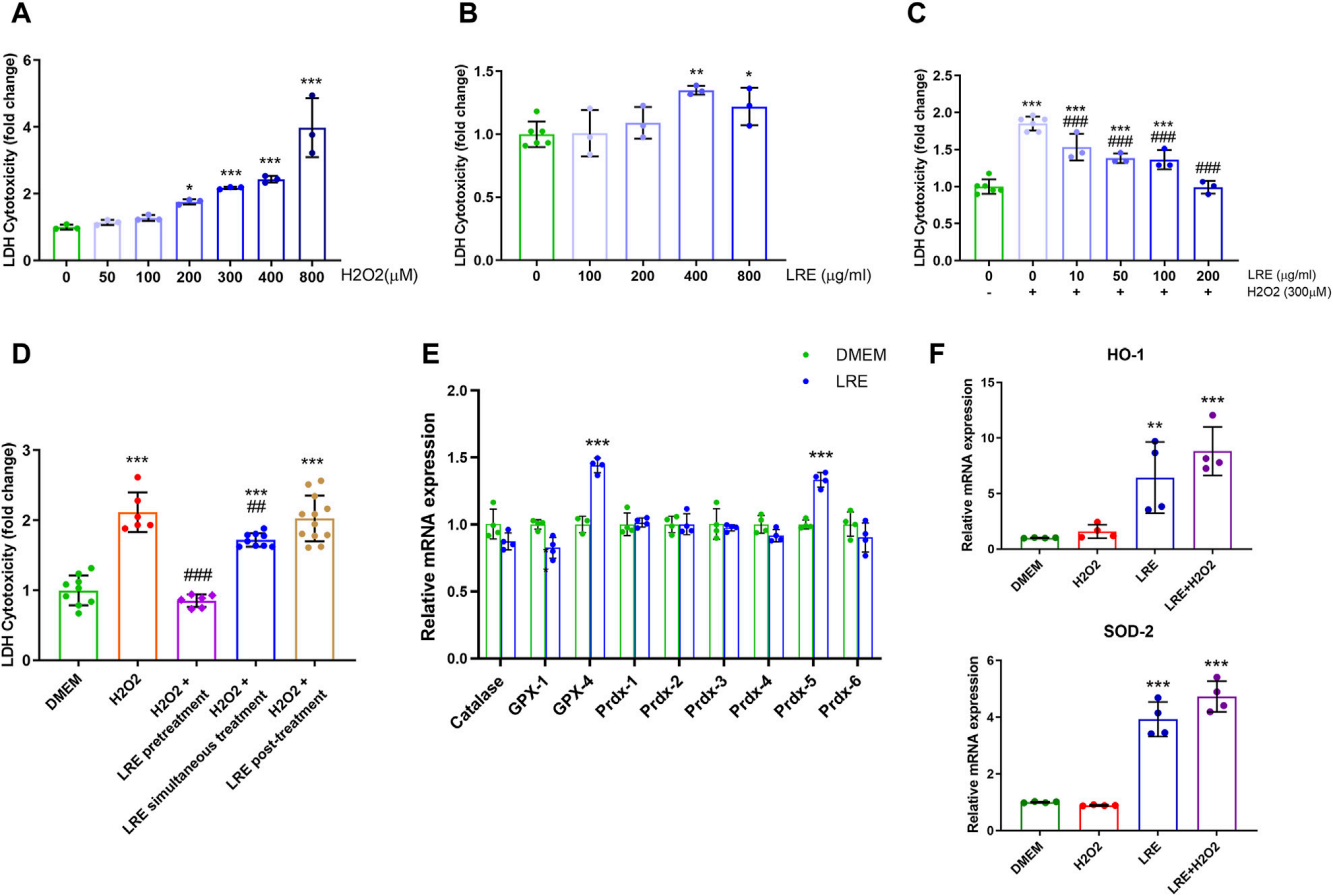
LRE protected IMG cells from oxidative stress by enhancing the expressions of antioxidant enzyme related genes. **(A)** Cytotoxicity assay of IMG cells treated by H2O2 at various concentrations, including 50, 100, 200, 300, 400, and 800 µM. **(B)** Cytotoxicity assay of IMG cells treated by LRE at various concentrations, including 100, 200, 400, and 800 μg/mL. **(C)** The effects of 2 h of pretreatment with LRE at 10, 50, 100, and 200 μg/mL on the cytotoxicity induced by 300 µM H2O2 in the IMG cells. **(D)** Pretreatment and simultaneous treatment but not post-treatment with 200 μg/mL LRE significantly reduced 300 µM H2O2-induced cytotoxicity in the IMG cells. **(E)** 2 h of 200 μg/mL LRE treatment on the IMG cells significantly upregulated the gene expressions of GPX-4 and Prdx-5. **(F)** Pretreatment with 200 μg/mL LRE significantly upregulated HO-1 and SOD-2 gene expressions in the IMG cells with or without the 300 µM H2O2 stimulation. *, *p* < 0.05; **, *p* < 0.01; ***, *p* < 0.001 vs. DMEM control. ##, *p* < 0.01; ###, *p* < 0.001 vs. H2O2 stimulation.

The antioxidation mechanisms of LRE were investigated by adding LRE to H2O2-treated IMG cells *in vitro*. H2O2 induced significant cytotoxicity in IMG cells in a concentration-dependent manner, starting from 200 μM. H2O2 at 300 μM increased cytotoxicity to IMG by 2.12 ± 0.28 folds than control (***p < 0.001, Figure 6A). Therefore, 300 μM was chosen to be the oxidative stress stimulator in the following experiments. LRE can also be a stressor to IMG cells. Below 200 μg/mL, there was no significant difference between LRE and no treatment control. However, when the LRE concentration increased to 400 and 800 μg/mL, there was significant cytotoxicity in IMG cells (Figure 6B). The protective effect of LRE was first evaluated by pretreating IMG cells with LRE from 10 to 200 μg/mL for 2 h and then challenging the cells with 300 μM H2O2. LRE significantly prevented H2O2 induced cytotoxicity in IMG cells in a concentration-dependent manner. 200 μg/mL LRE maintained IMG cell’s reaction at a level similar to no treatment control (Figure 6C). Following this, the best time for LRE application was further evaluated. 200 μg/mL LRE treatment at 2 h before H2O2 stimulation (pretreatment) significantly reduced the cytotoxicity to 0.85 ± 0.09 folds (***p < 0.001 vs. H2O2) and simultaneous administration of LRE with H2O2 also had a protective effect (**p = 0.003 vs. H2O2, Figure 6D). But when LRE was applied at 2 h after H2O2 stimulation (post-treatment), there was no protective effect.

The fact that pre- and simultaneous LRE treatment protected IMG cells from H2O2-induced oxidative stress, prompted the inquiry of whether LRE pretreatment can effectively prime microglial cells into an antioxidative status by enhancing enzymes involved in H2O2 decomposition, thereby enabling efficient cytoplasmic H2O2 elimination. Among the eight enzymes involved in H2O2 decomposition, GPX-4 and Prdx-5 were significantly increased to 1.44 ± 0.05 (vs. control, ***p < 0.001) and 1.33 ± 0.06 (vs. control, ***p < 0.001) folds, respectively, following LRE treatment for 2 h (Figure 6E). Furthermore, LRE pretreatment effects on the antioxidant genes HO-1 and SOD-2 were evaluated. While H2O2 demonstrated similar expression levels on these two genes to the control, 200 μg/mL LRE significantly increased the levels of HO-1 and SOD-2 to 6.43 ± 3.20 (vs. control, **p = 0.002) and 3.92 ± 0.61 folds (vs. control, ***p < 0.001), respectively. There was an accumulation effect of LRE pretreatment to H2O2 as shown in Figure 6F. The levels of HO-1 and SOD-2 raised to 8.80 ± 2.19 (vs. control, ***p < 0.001) and 4.73 ± 0.54 folds (vs. control, ***p < 0.001).

## 4 Discussion

Neuroprotection, antioxidation, and anti-inflammation effects of LR were summarized by Lee and Choi (2023). Our study investigated the effects of LRE in alleviating RGC degeneration in mouse models mimicking glaucoma. Daily LRE oral feeding significantly preserved RGC function, reduced apoptosis, and promoted RGC survival in the laser-induced COH mouse model and the DBA/2J mouse strain. LRE treatment lowered oxidative DNA damage of the retinal neurons especially in the RGC layer and the INL, as evidenced by reduced 8-OHdG expression. LRE reversed the increase of HO-1 and SOD-2 expressions in the retina of COH mice. Further *in vitro* study demonstrated that LRE pretreatment to IMG cells could significantly reduce H2O2 induced oxidative stress through upregulation of GPX-4, Prdx-5, HO-1 and SOD-2. Retinal microglial activation under sustained IOP elevation was reversed in the COH eyes but not the DBA/2J eyes.

Glaucoma is characterized by progressive RGC loss which leads to irreversible blindness. Current glaucoma treatment relies primarily on IOP lowering surgery/medication, however, was not enough to halt the disease progression (Saifi et al., 2023). Previous studies investigating the pathogenesis of glaucoma highlighted the critical contributions of oxidative stress and microglial activation (Wei et al., 2019; Fan Gaskin et al., 2021). IOP-independent neuroprotective treatments thus are warranted for the future development of glaucoma therapies (Jayaram et al., 2023). Neuroprotective treatments in conjunction with IOP lowering methods might slow down the disease progression especially if the diagnosis is confirmed at early stage. Two mouse models mimicking glaucoma were used in this study with different LRE treatment starting time. In the laser-induced COH model, the LRE oral feeding started at 7 days before the IOP increase. LRE was used as a preventative supplement aiming to potentiate the retinal resilience against high IOP. In the DBA/2J mice, the pigment dispersion in the anterior chamber was detectable from 5–6 months of age and became prominent at 9 months of age (Libby et al., 2005). The dispersed iris pigment obstructs the trabecular meshwork, resulting in secondary IOP elevation in DBA/2J eyes. Libby et al. (2005) found that IOP in DBA/2J eyes started to increase from 6 months of age, reached the highest level at 10 months of age, and then declined at 12 months of age. In this DBA/2J mice, oral feeding of the LRE started at 6 months of age indicating early interference in glaucoma. Oral taking of LRE at a dose of 2 g/kg improved the RGC function and survival without affecting the IOP elevation in both the COH and the DBA/2J mouse models.

LRE showed its antioxidant and anti-inflammatory effects in dextran sulfate sodium induced murine experimental colitis, exhaustive exercise-induced cardiac injury, high-fat diet-induced nonalcoholic fatty liver disease, and radiation injury (Duan et al., 2015; Lin et al., 2015; Hou et al., 2019; Lu et al., 2020; Zong et al., 2020). LRE administration was indicated to enhance the expressions of antioxidant enzymes such as SOD, GPX, and catalase in affected tissues, countering oxidative stress. In fact, anthocyanins, polyphenols, and polysaccharide from LR could activate the Nrf2/HO-1 signaling pathway which regulates a host of antioxidant enzymes (Deng et al., 2020; Tian et al., 2021; Gao et al., 2022). The anthocyanins and anthocyanidins in the LRE might be the key bioactive agents for the protective effect on RGCs under high IOP, they occupied 0.0416% (w/w). LRE oral feeding ameliorated the oxidative stress marked by 8-OHdG increase dominantly in the inner retina of the COH and the DBA/2J eyes. LRE pretreatment of IMG for 2 h successfully upregulated the H2O2 decomposition related enzyme GPX-4 and Prdx-1 gene expression. When H2O2 was added to the LRE primed IMG cells, antioxidant genes (HO-1 and SOD-2) expression increased even higher than the LRE treatment control group. Upregulated GPX-4, Prdx-1, HO-1, and SOD-2 in the LRE pretreatment group successfully prevented the H2O2 induced LDH increase to a level similar to the control group. While this *in vitro* finding is consistent with the reports in other systems, the finding in the COH model that HO-1 and SOD-2 returned to normal levels with LRE oral feeding was unexpected. We postulated that retinal tissue upregulated the antioxidant genes to combat oxidative stress induced by IOP elevation. Daily LRE intake, on the other hand, potentiated the antioxidant ability of retinal tissue, reducing ROS levels and restoring the retinal microenvironment to normalcy, obviating the need for further antioxidant gene increase. The inconsistency between our *in vivo* findings and others may stem from variations in pathological conditions and their temporal dynamics, which necessitates validation by further investigations.

The anti-inflammatory effect of LRE in the glaucoma models was evaluated by counting the Iba-1 positive cells in the retina. Elevated IOP caused significantly increased microglial activation. LRE as a pretreatment agent decreased Iba-1 positive cells in the COH eyes. In the IMG cell culture, the cytotoxicity of H2O2 can be prevented when LRE was applied as pre- or simultaneous but not post-treatment. In the DBA/2J mice, the RGC function started to be impaired from 3 months of age when the IOP remained at a normal level (Saleh et al., 2007; Harazny et al., 2009). Activation of retinal microglia in the DBA/2J mice is much earlier than 6 months when the LRE oral feeding started. LRE as a post-treatment did not reduce Iba-1 positive cells in the DBA/2J eyes. This differential effect of LRE on microglial activation under high IOP in COH and DBA/2J mice could be induced by the intricate genetic background and the considerable variation in disease progression (Turner et al., 2017).

We demonstrated that daily LRE feeding preserved the function of RGCs and enhanced their survival under the threat of sustained IOP elevation using two chronic glaucoma mouse models. This protective effect was likely attributed to reduced oxidative stress in the retinal neurons by LRE treatment, while inhibition of microglial activation could also contribute. *In vitro* study found that LRE pretreatment protected IMG cells from H2O2 induced damage by priming these microglial cells into an antioxidative status with upregulated GPX-4, Prdx-5, HO-1, and SOD-2. LRE may also confer neuroprotection to other retinal diseases such as retinitis pigmentosa and age-related macular degeneration in which oxidative stress in the ONL was featured (Murakami et al., 2020; Jabbehdari and Handa, 2021). LRE contains various bioactive components, such as anthocyanins, polyphenols, and polysaccharides. Future studies investigating the roles of specific LRE components in treating glaucoma would enhance the translation of LRE treatment to patients.

## 5 Conclusion

LRE oral feeding provided antioxidative effect, preserving the RGCs function and survival as a neuroprotective measure for glaucoma. The 4 months continuous oral feeding in DBA/2J mice that ended at 10 months of age is a precious indication for clinical application of LRE as a supplement to the current glaucoma treatment strategies which focus on IOP control.

## Data Availability

The original contributions presented in the study are included in the article/Supplementary Material, further inquiries can be directed to the corresponding authors.

## References

[B1] Allison K. Patel D. Alabi O. (2020). Epidemiology of glaucoma: the past, present, and predictions for the future. Cureus 12 (11), e11686. 10.7759/cureus.11686 33391921 PMC7769798

[B2] Andrés C. M. C. Lastra J. M. P. Juan C. A. Plou F. J. Pérez-Lebeña E. (2023). Chemical insights into oxidative and nitrative modifications of DNA. Int. J. Mol. Sci. 24 (20), 15240. 10.3390/ijms242015240 37894920 PMC10607741

[B3] Baudouin C. Kolko M. Melik-Parsadaniantz S. Messmer E. M. (2021). Inflammation in Glaucoma: from the back to the front of the eye, and beyond. Prog. Retin Eye Res. 83, 100916. 10.1016/j.preteyeres.2020.100916 33075485

[B4] Cui B. Liu L. Shi T. Yin M. Feng X. Shan Y. (2023). The ethanolic extract of Lycium ruthenicum ameliorates age-related physiological damage in mice. Molecules 28 (22), 7615. 10.3390/molecules28227615 38005337 PMC10673502

[B5] Deng K. Li Y. Xiao M. Wang F. Zhou P. Zhang W. (2020). Lycium ruthenicum Murr polysaccharide protects cortical neurons against oxygen-glucose deprivation/reperfusion in neonatal hypoxic-ischemic encephalopathy. Int. J. Biol. Macromol. 158, 562–568. 10.1016/j.ijbiomac.2020.04.122 32380112

[B6] Duan Y. Chen F. Yao X. Zhu J. Wang C. Zhang J. (2015). Protective effect of Lycium ruthenicum murr. Against radiation injury in mice. Int. J. Environ. Res. Public Health 12 (7), 8332–8347. 10.3390/ijerph120708332 26193298 PMC4515725

[B7] Fan Gaskin J. C. Shah M. H. Chan E. C. (2021). Oxidative stress and the role of NADPH oxidase in glaucoma. Antioxidants (Basel) 10 (2), 238. 10.3390/antiox10020238 33557289 PMC7914994

[B8] Feng L. Chen H. Suyeoka G. Liu X. (2013). A laser-induced mouse model of chronic ocular hypertension to characterize visual defects. J. Vis. Exp. 78, 50440. 10.3791/50440 PMC3846879 23979255

[B9] Fernández-Albarral J. A. Ramírez A. I. de Hoz R. Matamoros J. A. Salobrar-García E. Elvira-Hurtado L. (2024). Glaucoma: from pathogenic mechanisms to retinal glial cell response to damage. Front. Cell Neurosci. 18, 1354569. 10.3389/fncel.2024.1354569 38333055 PMC10850296

[B10] Gao H. Xue Y. Wu L. Huo J. Pang Y. Chen J. (2022). Protective effect of Lycium ruthenicum polyphenols on oxidative stress against acrylamide induced liver injury in rats. Molecules 27 (13), 4100. 10.3390/molecules27134100 35807346 PMC9267984

[B11] Harazny J. Scholz M. Buder T. Lausen B. Kremers J. (2009). Electrophysiological deficits in the retina of the DBA/2J mouse. Doc. Ophthalmol. 119 (3), 181–197. 10.1007/s10633-009-9194-5 19760280

[B12] Hou C. W. Chen I. C. Shu F. R. Feng C. H. Hung C. T. (2019). Protective effect of supplementation with Lycium ruthenicum Murray extract from exhaustive exercise-induced cardiac injury in rats. Chin. Med. J. Engl. 132 (8), 1005–1006. 10.1097/CM9.0000000000000185 30958451 PMC6595767

[B13] Jabbehdari S. Handa J. T. (2021). Oxidative stress as a therapeutic target for the prevention and treatment of early age-related macular degeneration. Surv. Ophthalmol. 66 (3), 423–440. 10.1016/j.survophthal.2020.09.002 32961209

[B14] Jayaram H. Kolko M. Friedman D. S. Gazzard G. (2023). Glaucoma: now and beyond. Lancet 402 (10414), 1788–1801. 10.1016/S0140-6736(23)01289-8 37742700

[B15] Lee H. S. Choi C. I. (2023). Black goji berry (Lycium ruthenicum Murray): a review of its pharmacological activity. Nutrients 15 (19), 4181. 10.3390/nu15194181 37836464 PMC10574788

[B16] Libby R. T. Anderson M. G. Pang I. H. Robinson Z. H. Savinova O. V. Cosma I. M. (2005). Inherited glaucoma in DBA/2J mice: pertinent disease features for studying the neurodegeneration. Vis. Neurosci. 22 (5), 637–648. 10.1017/S0952523805225130 16332275

[B17] Lin J. Zhang Y. Wang X. Wang W. (2015). Lycium ruthenicum extract alleviates high-fat diet-induced nonalcoholic fatty liver disease via enhancing the AMPK signaling pathway. Mol. Med. Rep. 12 (3), 3835–3840. 10.3892/mmr.2015.3840 26017330

[B18] Liu Z. Tang X. Liu C. Dong B. Shao Y. Liu B. (2020). Ultrasonic extraction of anthocyanins from Lycium ruthenicum Murr. and its antioxidant activity. Food Sci. Nutr. 8 (6), 2642–2651. 10.1002/fsn3.1542 32566181 PMC7300067

[B19] Lu K. Wang J. Yu Y. Wu Y. He Z. (2020). Lycium ruthenicum Murr. alleviates nonalcoholic fatty liver in mice. Food Sci. Nutr. 8 (6), 2588–2597. 10.1002/fsn3.1445 32566176 PMC7300084

[B20] McMonnies C. (2018). Reactive oxygen species, oxidative stress, glaucoma and hyperbaric oxygen therapy. J. Optom. 11 (1), 3–9. 10.1016/j.optom.2017.06.002 28760643 PMC5777925

[B21] Murakami Y. Nakabeppu Y. Sonoda K. H. (2020). Oxidative stress and microglial response in retinitis pigmentosa. Int. J. Mol. Sci. 21 (19), 7170. 10.3390/ijms21197170 32998461 PMC7583782

[B22] Pang Y. Chen J. Yang J. Xue Y. Gao H. Gao Q. (2023). Protective effect and mechanism of Lycium ruthenicum polyphenols against acrylamide-induced neurotoxicity. Food Funct. 14 (10), 4552–4568. 10.1039/d3fo00623a 37021634

[B23] Saifi A. I. Nagrale P. Ansari K. K. Saifi I. Chaurasia S. (2023). Advancement in understanding glaucoma: a comprehensive review. Cureus 15 (9), e46254. 10.7759/cureus.46254 37908941 PMC10614105

[B24] Saleh M. Nagaraju M. Porciatti V. (2007). Longitudinal evaluation of retinal ganglion cell function and IOP in the DBA/2J mouse model of glaucoma. Invest. Ophthalmol. Vis. Sci. 48 (10), 4564–4572. 10.1167/iovs.07-0483 17898279 PMC2765717

[B25] Simpson D. S. A. Oliver P. L. (2020). ROS generation in microglia: understanding oxidative stress and inflammation in neurodegenerative disease. Antioxidants (Basel) 9 (8), 743. 10.3390/antiox9080743 32823544 PMC7463655

[B26] Tian B. Zhao J. Xie X. Chen T. Yin Y. Zhai R. (2021). Anthocyanins from the fruits of Lycium ruthenicum Murray improve high-fat diet-induced insulin resistance by ameliorating inflammation and oxidative stress in mice. Food Funct. 12 (9), 3855–3871. 10.1039/d0fo02936j 33704297

[B27] Turner A. J. Vander Wall R. Gupta V. Klistorner A. Graham S. L. (2017). DBA/2J mouse model for experimental glaucoma: pitfalls and problems. Clin. Exp. Ophthalmol. 45 (9), 911–922. 10.1111/ceo.12992 28516453

[B28] Wang H. Li J. Tao W. Zhang X. Gao X. Yong J. (2018). Lycium ruthenicum studies: molecular biology, Phytochemistry and pharmacology. Food Chem. 240, 759–766. 10.1016/j.foodchem.2017.08.026 28946340

[B29] Wang X. Wang M. Liu H. Mercieca K. Prinz J. Feng Y. (2023). The association between vascular abnormalities and glaucoma-what comes first? Int. J. Mol. Sci. 24 (17), 13211. 10.3390/ijms241713211 37686017 PMC10487550

[B30] Wei X. Cho K. S. Thee E. F. Jager M. J. Chen D. F. (2019). Neuroinflammation and microglia in glaucoma: time for a paradigm shift. J. Neurosci. Res. 97 (1), 70–76. 10.1002/jnr.24256 29775216 PMC6239948

[B31] Zong S. Yang L. Park H. J. Li J. (2020). Dietary intake of Lycium ruthenicum Murray ethanol extract inhibits colonic inflammation in dextran sulfate sodium-induced murine experimental colitis. Food Funct. 11 (4), 2924–2937. 10.1039/d0fo00172d 32285052

